# Longitudinal associations between white matter integrity, early life adversities, and treatment response following cognitive-behavioral therapy in depression

**DOI:** 10.1038/s41386-025-02070-x

**Published:** 2025-02-26

**Authors:** Kira Flinkenflügel, Tiana Borgers, Melissa Klug, Marie M. Mummendey, Elisabeth J. Leehr, Susanne Meinert, Marius Gruber, Jonathan Repple, Tilo Kircher, Nils Opel, Jochen Bauer, Esther Zwiky, Philine König, Antonia Küttner, Konrad Schöniger, Robin Kamrla, Udo Dannlowski, Verena Enneking, Ronny Redlich

**Affiliations:** 1 Institute for Translational Psychiatry, University of Münster, Münster, Germany; 2 Institute for Translational Neuroscience, University of Münster, Münster, Germany; 3 Department of Psychiatry, Psychosomatic Medicine and Psychotherapy, University Hospital Frankfurt, Goethe University, Frankfurt, Germany; 4 Department of Psychiatry and Psychotherapy, University of Marburg, Marburg, Germany; 5 Center for Mind, Brain and Behavior (CMBB), University of Marburg, Marburg, Germany; 6 Department of Psychiatry and Psychotherapy, University Hospital Jena, Jena, Germany; 7 German Center for Mental Health (DZPG), Halle-Jena-Magdeburg, Halle, Germany; 8 Department of Radiology, University of Münster, Münster, Germany; 9 Department of Psychology, University of Halle, Halle, Germany; 10 Center for Intervention and Research on Adaptive and Maladaptive Brain Circuits Underlying Mental Health (C-I-R-C), Halle-Jena-Magdeburg, Halle, Germany

**Keywords:** Depression, Depression, Predictive markers, Risk factors

## Abstract

Cognitive-behavioral therapy (CBT) is a primary treatment for depression. Although previous research has underscored the significant roles of white matter (WM) alterations and maladaptive parenting in depression risk, their associations with CBT response remain largely unknown. This longitudinal study investigated the interplay of WM integrity changes over time, treatment response, and parenting style in patients with depression. Diffusion-tensor-imaging and clinical data were assessed in *n* = 65 (55% female) patients with depression before and after 20 CBT sessions and *n* = 65 (68% female) healthy controls (HC) in a naturalistic design. Linear-mixed-effect models compared changes in fractional anisotropy (FA) between groups and tested associations between FA changes and symptom changes. It was investigated whether parenting style predicts depressive symptoms at follow-up and whether FA changes mediate this association. Patients showed differential FA changes over time in the corpus callosum and corona radiata compared to HC (*p*_tfce-FWE_ = 0.008). Increases in FA in the corpus callosum, corona radiata and superior longitudinal fasciculus were linked to symptom improvement after CBT in patients (*p*_tfce-FWE_ = 0.023). High parental care (*p*_FDR_ = 0.010) and low maternal overprotection (*p*_FDR_ = 0.001) predicted fewer depressive symptoms at follow-up. The association between maternal overprotection and depressive symptoms at follow-up was mediated by FA changes (*p*_FDR_ = 0.044). Robustness checks—controlling for outliers, non-linear age effects, clinical characteristics, and patient subgroups—supported these results. Overall, patients with depression show changes in WM integrity following CBT, which are linked to treatment response. The results highlight the significance of early life adversities and related microstructural changes in the effectiveness of CBT for treating depression.

## Introduction

Depression is one of the most prevalent mental diseases worldwide, with an estimated lifetime prevalence of 17% [1]. Depression is characterized by chronicity and recurrence, leading to severe impairment in social and occupational domains [2]. Cognitive-behavioral therapy (CBT) is a first-line psychotherapeutic treatment for depression that focuses on the identification and modification of dysfunctional cognitions and behaviors [3]. While previous studies have demonstrated the superiority of CBT compared to other antidepressant treatments in the long-term [4], there is still a substantial number of patients who do not benefit from CBT [5, 6]. Investigating the neural underpinnings of CBT and determining predictors of treatment outcome might help to improve our understanding of CBT and optimize antidepressant treatment options.

Neuroimaging research in depression has demonstrated microstructural alterations in white matter (WM) pathways responsible for connecting and transferring information between different regions of the human brain. Fractional anisotropy (FA) is a widely used measure in diffusion tensor imaging (DTI) that reflects the degree of directionality in water diffusion within white matter. Higher FA values generally indicate greater alignment of axonal fibers along the longitudinal axis and suggest intact microstructural organization, including myelin integrity. Cross-sectional DTI studies have identified lower FA in patients with depression compared to healthy controls (HC) in the corpus callosum, superior longitudinal fasciculus, and corona radiata [7, 8], with alterations most pronounced in individuals suffering from recurrent depression [9, 10]. To date, only few longitudinal DTI studies in patients with depression have been conducted [11–13], yielding a rather inconsistent pattern of results. However, recent evidence suggests that patients with depression show reductions in FA compared to HC over a 2-year follow-up interval [14]. Regarding antidepressant treatment, prospective DTI studies have revealed increases [15–18] and decreases [19, 20] of WM microstructural integrity after electroconvulsive or psychopharmacological therapy, with different DTI metrics associated with treatment response [16, 17, 20, 21]. Despite the high clinical relevance of CBT in treating depression, no DTI study has yet investigated the microstructural underpinnings of CBT and their associations with treatment response in depression.

Treatment response and microstructural correlates might be influenced by several environmental factors. For instance, childhood experiences of parental neglect and overprotection are important risk factors for the development and maintenance of depression [22–24]. Studies have reported that low parental care and high parental overprotection are associated with more depressive symptoms [25, 26] and severe disease courses [23, 27]. Moreover, these factors may also correlate with a lower likelihood of remission following antidepressant treatment [23, 28]. For example, Asano and colleagues demonstrated that high maternal overprotection is associated with a lower response to psychotherapy [28]. However, this finding was limited by small sample sizes and lacking correction for clinical measures (e.g., current psychiatric medication, comorbidities, previous disease course before treatment). At the biological level, poor parenting style has been linked to altered cortisol response [29, 30] and lower FA in healthy individuals [31]. This interplay between disease progression, parenting style, and WM microstructural integrity may potentially affect responsiveness to antidepressant treatments such as CBT and warrants investigation.

This prospective and naturalistic DTI study aimed to investigate the longitudinal associations between parenting style, changes in WM microstructural integrity, and treatment response following CBT in patients with depression. To this end, longitudinal changes in FA in patients with depression, receiving CBT during the interval, compared to HC were examined at the whole-brain level (analysis 1). Based on the heterogeneity of previous longitudinal findings, it was hypothesized that patients show differential changes in FA in the corpus callosum, superior longitudinal fasciculus, and corona radiata over time compared to HC. Furthermore, associations between FA changes and symptom changes after CBT in patients with depression were tested (*analysis 2*), with an increase in FA expected to be associated with symptom improvement after CBT. Lastly, the role of parenting style in the relationship between FA and CBT response in patients was investigated (*analysis 3*). Consistent with previous studies, it was assumed that better (i.e., more caring and less controlling) parenting style predicts fewer depressive symptoms after CBT and that this association is mediated by FA changes.

## Materials and methods

### Participants

This prospective and naturalistic study included *n* = 65 HC and *n* = 65 patients with depression from the ongoing study of the Prevention and Intervention Neuroimaging Cohort (PINC) (Table 1, Supplement 1). All patients received CBT during the study interval and were measured shortly before treatment initiation (i.e., waiting list or trial phase; baseline) and after approximately 20 sessions (*M* = 20.83, *SD* = 3.70) of naturalistic CBT (excluding the trial phase; follow-up) (Supplement 2). HC underwent assessments at equivalent time points (interscan interval in months: *M*_patients_ = 8.09, *SD*_patients_ = 2.30; *M*_HC_ = 8.19, *SD*_HC_ = 1.72; *p* = 0.782). At both time points, participants completed a DTI measurement, a clinical interview and the Hamilton Depression Rating Scale (HDRS, [32]) to assess current depressive symptoms. At baseline, the Parental Bonding Instrument (PBI) was used, which evaluates participants’ perceptions of their caregivers’ parenting styles (four scores: maternal care, paternal care, maternal overprotection, paternal overprotection) before age 16, with higher scores reflecting higher parental care or higher overprotection [33]. Further, patients were included if they fulfilled criteria for an acute or partially remitted major depressive disorder, acute dysthymia or acute adjustment disorder with depressed mood. HC were included if they had no history of mental disorders. Psychiatric diagnoses were determined using the Structured Clinical Interview for the Diagnostic and Statistical Manual of Mental Disorders, Version 4, Text Revision (DSM-IV-TR) for Axis I disorders (SCID-I) by trained raters [34]. Patients were recruited from the psychotherapeutic outpatient unit of the University of Muenster between August 18, 2017, and September 26, 2022. HC were recruited through public notices and newspaper advertisements. The study received approval from the ethics committee of the medical faculty of the University of Münster (Amendment of 2016-173-f-S; 2020-205-f-S), and all experiments and procedures adhered to ethical guidelines and regulations. Written informed consent was obtained from all participants, who also received financial compensation.

**Table 1:** Sociodemographic and clinical characteristics of the sample.

	Patients with depression *n* = 65	Healthy controls *n* = 65	*p* value^a^	Effect size^b^
	Mean (SD)	Mean (SD)		
**Sociodemographic characteristics**				
Age at baseline (years)	26.86 (7.26)	38.06 (17.20)	**<0.001**	0.849
Sex (m/f), No. of patients (%)^c^	29 (45)/36 (55)	21 (32)/44 (68)	0.149	0.126
Interscan interval (days)	246.31 (70.21)	249.32 (52.44)	0.782	0.049
**Symptom severity**				
HDRS at baseline	12.20 (6.27)	1.77 (2.58)	**<0.001**	2.174
HDRS at follow-up^d^	7.29 (5.76)	1.00 (1.53)	**<0.001**	1.488
**Parenting style**				
Maternal care^e^	24.17 (8.10)	28.60 (7.64)	**<0.001**	0.563
Maternal overprotection^e^	12.95 (7.78)	8.91 (6.58)	**<0.001**	0.562
Paternal care^f^	20.34 (8.86)	25.90 (9.29)	**<0.001**	0.613
Paternal overprotection^f^	8.83 (6.47)	7.45 (5.76)	0.217	0.225
**Clinical characteristics at baseline**				
Diagnosis (MDD/ dysthymia/ adjustment disorder with depressed mood), No. of patients (%)	59 (91)/1 (1)/5 (8)	–	–	–
Disease progression of MDD (*n* = 59) before baseline (first episode/ recurrent), No. of patients (%)	22 (37)/37 (63)	–	–	–
No. of depressive episodes before baseline^g^	2.33 (2.05)	–	–	–
Cumulative duration of depressive episodes before baseline (months)^h^	33.19 (42.34)	–	–	–
Age of onset (years)^g^	20.95 (7.49)	–	–	–
Remission status (no/ partial/ full remission), No. of patients (%)	47 (72)/18 (28)/0 (0)	–	–	–
**Clinical characteristics at follow-up**				
No. of CBT sessions between t_0_ and t_1_	20.83 (3.70)	–	–	–
Remission status (no/ partial / full remission), No. of patients (%)	14 (22)/32 (49)/19 (29)	–	–	–
**Comorbidity**				
Acute comorbidity (yes/no), No. of patients at baseline (%)	28 (43)/37 (57)	–	–	–
Acute comorbidity (yes/no), No. of patients at follow-up (%)	14 (22)/51 (78)	–	–	–
**Psychopharmacological treatment**				
Medication load index at baseline	0.51 (0.87)	–	–	–
Medication load index at follow-up	0.55 (0.88)	–	–	–

*HDRS* Hamilton depression rating scale, *BDI* Beck depression inventory, *MDD* major depressive disorder.

^a^*p* values were obtained using the unpaired two-tailed *t* test except where noted.

^b^Effect sizes were obtained using Cohen’s d except where noted.

^c^*p* values were obtained using the χ^2^-test. Effect sizes were obtained using Phi.

^d^Information was missing for *n* = 1 in the healthy control group.

^e^Information was missing for *n* = 1 in the patient group.

^f^Information was missing for *n* = 3 in the patient group and *n* = 5 in the HC group.

^g^Information was missing for *n* = 2 in the patient group.

^h^Information was missing for *n* = 3 in the patient group.

### DTI preprocessing

DTI data were measured using a 3 T magnetic resonance imaging (MRI) scanner (Prisma, Siemens, Erlangen, Germany). Preprocessing was performed in FSL6.0.1 (http://fsl.fmrib.ox.ac.uk/fsl/fslwiki/, FMRIB, Oxford Center for Functional MRI of the Brain, University of Oxford, Department of Clinical Neurology, John Radcliffe Hospital, Oxford, United Kingdom) [35–37], using a longitudinal preprocessing stream (Supplement 3, Supplement 4) While this study focuses on FA, results on further diffusion metrics are reported in Supplement 5.

### Statistical analyses

Demographic and clinical data were analyzed using R studio (version 4.0.2; R Core Team, 2020). To investigate the influence of CBT on the severity of depressive symptoms, a random intercepts fixed slopes linear mixed-effect (LME) model was performed in patients with HDRS as outcome variable and time (baseline vs. follow-up) as predictor variable, while correcting for age. Fixed slopes were used for all LME models, as the inclusion of random slopes did not result in a significant improvement in model fit (Supplement 6). Furthermore, for LME models, we did not control for variables that do not change between measurements, as these are already considered by the random intercepts of participants. Neuroimaging analyses were conducted voxel-wise at the whole-brain level using tract-based-spatial-statistics (TBSS, correction: TFCE, 5000 permutations for *p* < 0·05 FWE-correction, two-sided when based on non-directional hypotheses and one-sided when based on directional hypotheses) [38]. While tractography-based methods approaches have allowed for tract reconstruction at the subject level [39], we chose TBSS as it is a widely accepted tool [8] and offers a fully automated voxel-wise analysis of the whole brain. This approach minimizes the risk of overlooking patterns or relationships beyond predefined regions. In contrast, region of interest-based approaches are limited by potential biases in prior region selection [40, 41].

### *Analysis 1*: differences in FA changes between patients with depression and HC

To test whether FA changes over time differ between patients versus HC, a random intercepts fixed slopes LME model was conducted in FSL with FA as outcome variable and time (baseline vs. follow-up) and diagnosis (HC vs. depression) as predictor variables, while controlling for total intracranial volume (TIV) and age. The main effect of time and the diagnosis×time interaction were analyzed. Since the LME model in FSL does not provide meaningful cross-sectional contrasts, cross-sectional group analyses for baseline and follow-up data were conducted with separate general linear models.

### *Analysis 2*: FA changes and symptom changes after CBT

To analyze the association between FA changes and symptom changes after CBT, another random intercepts fixed slopes LME model was performed in patients with FA as outcome variable and time as well as HDRS as predictor variables, controlling for TIV and age. The HDRS×time interaction was analyzed. As an exploratory approach, it was further investigated whether responders (i.e., patients who achieved full or partial remission after CBT) and non-responders (i.e., patients who were still acutely depressed after CBT) differed in 1) longitudinal FA changes over the interval; and 2) cross-sectional FA at baseline or follow-up. Therefore, a random intercepts fixed slopes LME model was calculated with FA as outcome variable, time and response (non-responders vs. responders) as predictor variables, TIV, age, and remission status at baseline as covariates. The response×time interaction was investigated. For cross-sectional analyses, two separate general linear models were calculated (one for baseline and one for follow-up) with FA from each timepoint as outcome variable, response as predictor variable, and TIV, age, sex, and remission status at baseline as covariates. The main effect of response was investigated for each model. See Supplement 7 for a detailed description of the definition of remission status.

### *Analysis 3*: parenting style, FA changes, and symptom severity after CBT

To test whether parenting style is associated with symptom changes after CBT and whether this association is mediated by FA changes, mediation analyses were performed in patients using a bootstrapping approach implemented in the macro PROCESS (http://www.processmacro.org). Parenting style was entered as predictor variable, mean ΔFA derived from significant clusters of *analysis 2* as mediator, and HDRS at follow-up as outcome variable into the model. Separate mediation models were calculated for each of the four PBI scores, with false discovery rate (FDR) correction applied to correct for multiple testing. HDRS_baseline_, TIV_baseline_, ΔTIV, age_baseline_, and interscan interval were included as covariates.

## Results

HDRS scores significantly decreased in patients from baseline to follow-up (*t*_(1,63)_ = -5.62, *p* < .001, *sr*^2^ = 0.143), indicating symptom improvement after CBT. *N* = 51 (78.5%) patients achieved full or partial remission (responders) and *n* = 14 (21.5%) patients remained in an acute depressive state (non-responders).

### *Analysis 1*: FA changes between patients with depression and HC

**Fig. 1:**
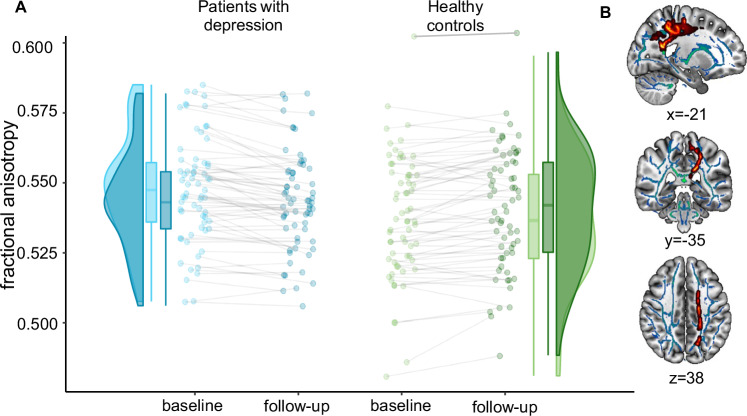
Longitudinal group differences in fractional anisotropy. **A** This figure shows the distribution, means, individual measurements, and slopes of FA, derived from the significant *t*-contrast, for patients with depression and HC at baseline and follow-up. Each dot represents one participant. Patients with depression showed more decline in FA than HC over time. **B** Interaction effect of time and diagnosis. Mean FA values of the significant clusters were extracted using ‘fslstats’ in FSL and displayed onto the SPM152 template in the x = −-21, y = –35, z = 38 plane in MNI space using MRIcroGL. Red-yellow represents voxels, in which a significant interaction effect was found. FA fractional anisotropy.

The analysis revealed a significant diagnosis×time interaction in the corpus callosum and corona radiata (*p*_tfce-FWE_ = .006, *sr*^2^ = 0.010, k = 1445 voxel in 2 clusters, Supplement 8, Supplementary Tables 1–2). Post-hoc-*t*-contrasts showed that patients showed greater decreases in FA following CBT compared to HC (*p*_tfce-FWE_ = .008, *sr*^2^ = 0.008, k = 2999 voxel in 1 cluster, Fig. 1, Supplement 8, Supplementary Tables 1–2). There was no significant main effect of time (*p*_tfce-FWE_ = .532). Neither at baseline (*p*_tfce-FWE_ = .576), nor at follow-up (*p*_tfce-FWE_ = .314), could cross-sectional group differences be observed.

### *Analysis 2*: FA changes and symptom changes after CBT

**Fig. 2:**
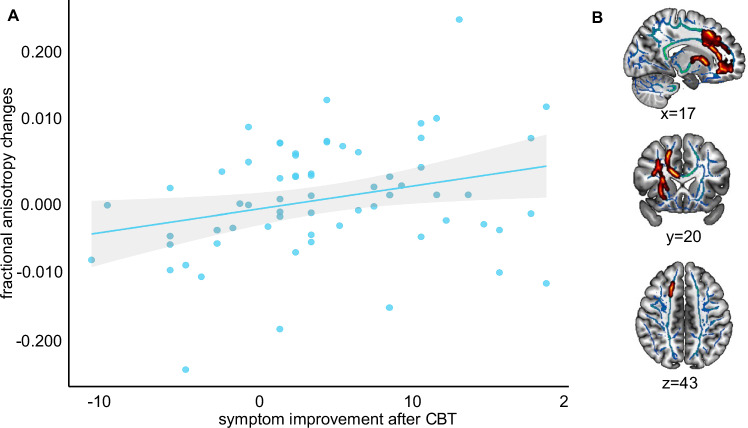
Longitudinal association between depressive symptom changes and fractional anisotropy changes in patients with depression. **A** Scatterplot depicting the longitudinal association between symptom improvement, measured with the Hamilton Depression Rating Scale (HDRS) and FA changes derived from the significant *t*-contrast in patients with depression. Symptom improvements over time were positively associated with FA changes. **B** Interaction effect of time and symptom improvement. Mean FA values of the significant clusters were extracted using ‘fslstats’ in FSL and displayed onto the SPM152 template in the *x* = 17, *y* = 20, *z* = 43 plane in MNI space using MRIcroGL. Red-yellow represents voxels, in which a significant interaction effect was found. CBT cognitive behavioral therapy, FA fractional anisotropy.

The analysis of FA and symptom changes after CBT yielded a significant HDRS×time interaction in the corpus callosum, superior longitudinal fasciculus, and corona radiata: Symptom improvement after CBT was associated with increases in FA after CBT (*p*_tfce-FWE_ = .023, *sr*^2^ = 0.034, k = 4741 voxel in 1 cluster, Fig. 2, Supplement 8, Supplementary Tables 1–2). The exploratory analysis revealed a significant response×time interaction in the corpus callosum and corona radiata: responders showed greater increase in FA over time compared to non-responders (*p*_tfce-FWE_ = .016, *sr*^2^ = 0.012, k = 614 voxel in 1 cluster, Supplement 8, Supplementary Tables 1–2). Furthermore, responders compared to non-responders showed higher FA at baseline in the corpus callosum, corona radiata, and superior longitudinal fasciculus (*p*_tfce-FWE_ = .020, *sr*^2^ = 0.255, k = 1165 voxel in 1 cluster, Supplement 8, Supplementary Tables 1–2) as well as follow-up in widespread fiber tracts, mainly affecting the corpus callosum, corona radiata, and superior longitudinal fasciculus (*p*_tfce-FWE_ = 0.004, *sr*^2^ = 0.407, k = 24271 voxel in 1 cluster, Supplement 8, Supplementary Tables 1–2).

### *Analysis 3*: parenting style, FA changes, and symptom severity after CBT

**Fig. 3:**
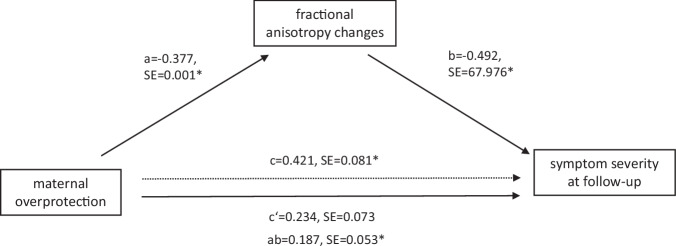
Fractional anisotropy changes mediated the link between maternal overprotection and depressive symptoms at follow-up in patients. The figure depicts the mediator model with maternal overprotection as the predictor variable, fractional anisotropy changes (follow-up-baseline) as the mediator variable, and depressive symptom severity at follow-up as the outcome variable in patients with depression. standardized coefficients and standard errors for each path of the mediation model are presented. Note that c represents the total effect, c‘ the direct effect, and ab the indirect effect. *indicates significance at *p*_FDR_ < 0.05.

The four mediation analyses within patients showed a negative association between HDRS at follow-up and maternal care (total effect: *β* = –0.316, SE = 0.079, *t* = –2.77, *p*_FDR_ = 0.010) as well as paternal care (total effect: *β* = -0.354, SE = 0.083, *t* = –2.77, *p*_FDR_ = 0.010), and a positive association between HDRS at follow-up and maternal overprotection (total effect: *β* = 0.421, SE = 0.081, *t* = 3.74, *p*_FDR_ = 0.001), but not paternal overprotection (total effect: *p*_FDR_ = 0.989). This indicates that high maternal and paternal care as well as low maternal overprotection prospectively predict fewer depressive symptoms at follow-up. ΔFA was also negatively associated with HDRS at follow-up (maternal care: *β* = –0.531, SE = 65.881, *t* = –5.53, *p*_FDR_ < 0.001; paternal care: *β* = –0.586, SE = 59.706, *t* = –6.63, *p*_FDR_ < 0.001; maternal overprotection: *β* = –0.492, SE = 67.976, *t* = –4.97, *p*_FDR_ < 0.001; paternal overprotection: *β* = –0.623, SE = 64.164, *t* = –6.55, *p*_FDR_ < 0.001), indicating that FA increases over time are associated with fewer depressive symptoms at follow-up. Furthermore, a significant positive indirect (mediated) effect of maternal overprotection on HDRS at follow-up through ΔFA (*β* = 0.187, SE = 0.053, *p*_FDR_ = 0.044, 95%-CI [0.041, 0.326], Fig. 3) was observed. The direct effect of maternal overprotection on HDRS at follow-up was not significant (*β* = 0.234, SE = 0.073, *p*_FDR_ = 0.050, *p*_uncorrected_ = 0.025, 95%-CI [0.021, 0.318]), indicating that the association between maternal overprotection and depressive symptoms at follow-up tended to be fully mediated by FA changes. No indirect (mediated) effect of ΔFA was found for the associations between maternal care (95%-CI [-0.274, 0.027]), paternal care (95%-CI [-0.212, 0.112]), or paternal overprotection (95%-CI [–0.073, 0.287]) with HDRS at follow-up.

### Robustness checks

Several robustness checks (Supplement 9, Supplementary Tables 3–17) for main results were conducted, including the correction for outliers, non-linear age effects, additional covariates at baseline and clinical characteristics in patients (e.g., current medication adherence, comorbidities, previous disease course). Furthermore, analyses were repeated excluding patients suffering from acute dysthymia or acute adjustment disorder at baseline (*n* = 6). Overall, robustness checks revealed a comparable pattern of results.

## Discussion

This longitudinal study investigated changes in WM microstructural integrity in patients with depression undergoing naturalistic CBT relative to HC, as well as the link of these changes with CBT response and parenting style. Patients and HCs experienced differential changes in FA over time, with patients showing reductions in FA in the corpus callosum and corona radiata, fiber tracts commonly affected in depression [8, 42, 43]. Notably, these FA reductions were mainly driven by patients not responding to CBT. In contrast, increases in FA were associated with symptom improvement after CBT. Moreover, responders had higher FA both before and after CBT compared to non-responders. Finally, high levels of parental care as well as low levels of maternal overprotection predicted fewer depressive symptoms after CBT. The association between low maternal overprotection and fewer depressive symptoms was mediated by FA changes. Robustness checks, including corrections for outliers, non-linear age effects, clinical characteristics, and the exclusion of specific patient subgroups, consistently revealed a comparable pattern of results, underscoring the reliability of the findings.

Aligning with prior research documenting declines in FA in patients with depression during the disease course compared to HC [11, 14], this study observed a significant decrease in FA in patients during CBT. While this finding may appear counterintuitive, similar inconsistencies in WM microstructural integrity changes have been noted in studies on antidepressant treatments [15–20]. One possible explanation is that the cumulative effects of prolonged depression and chronic stress, a key factor in the pathophysiology of depression [44, 45], prior to treatment, characterized by heightened hypothalamic-pituitary-adrenal axis activity and elevated glucocorticoid levels, may have precipitated neurodegeneration [44] and compromised WM fiber integrity [46, 47]. These detrimental effects could still be detectable as a delayed consequence during CBT, despite the intervention’s focus on reducing stress-inducing thought patterns.

At the same time, further analyses revealed that symptom improvement following CBT correlates with FA increases within the corpus callosum, superior longitudinal fasciculus, and corona radiata among patients with depression. This suggests that the overall FA decreases in patients compared to HC might be largely driven by the CBT non-responders, as demonstrated by our exploratory analysis comparing responders versus non-responders. Alternatively, the coexistence of responders and non-responders, each exhibiting distinct trajectories in WM integrity, may underlie the net FA decrease, thereby complicating the interpretation of these group-level results. Future research should investigate these subgroup effects more thoroughly to clarify the interplay between WM microstructure changes, chronic stress, and differential treatment responses. Observations of increased FA associated with CBT response suggest enhanced fiber coherence and organization [17]. The anterior corona radiata includes fibers that link limbic, thalamic and prefrontal regions [18, 48], crucial for emotion and cognitive processing [49–51]. Additionally, the superior longitudinal fasciculus plays a role in modulating the dorsolateral prefrontal cortex [52, 53]. Disruptions in WM connectivity may contribute to diminished top-down cognitive control, possibly leading to rumination [54] and emotion regulation deficits [49]. Reduced corpus callosum volume has been linked to diminished working memory capacity and processing speed [55], mirroring cognitive disruptions observed in depression [56]. Consequently, increases in FA of these fiber tracts may reflect improvements in top-down emotion and cognitive processing, thereby potentially highlighting their relevance for improvements in fronto-limbic dysfunctions [57–59] and the antidepressant mechanism. These results cautiously suggest that FA decreases may be attributed to the course of the disease of depression, while FA increases could indicate a positive effect of CBT in those responding to treatment. Additionally, responders showed higher baseline FA compared to non-responders, raising the speculation that pre-treatment FA could play a role in predicting treatment response. To determine whether these differential FA changes during CBT are ultimately attributable to the therapy itself and/or the progression of the disease over time, an untreated patient control group is essential in future research. Furthermore, while baseline FA shows potential as a predictive marker, individualized prediction requires the application of machine learning approaches in larger, multicenter studies.

In line with previous studies showing a link between poor parenting style and lower pharmacotherapy [23, 60] and CBT [28] responses, the findings demonstrate that high maternal and paternal care and low maternal overprotection are predictive of fewer depressive symptoms after CBT. This observation may be attributed to the development of secure attachments during childhood, potentially enabling individuals to develop effective emotion regulation strategies and robust coping mechanisms against adversities [61–63]. Such factors may likely enhance the efficacy of CBT, potentially augmenting responsiveness to techniques such as cognitive restructuring [61]. Additionally, a balance of high parental care and low overprotection could foster autonomy and self-efficacy [64], contributing to lower psychological distress [63] and serving as a protective factor against depressive symptoms [65, 66]. Asano and colleagues [28] also highlight the relevance of the therapeutic relationship in CBT’s success [67–70], suggesting that less controlling parenting styles may foster better therapeutic relationships, thereby improving treatment outcome.

Crucially, the relationship between low maternal overprotection and fewer depressive symptoms following CBT was mediated by changes in FA. These novel findings suggest that high maternal overprotection may result in decreases in WM microstructural integrity, subsequently impacting the depressive symptom severity after CBT and thereby CBT efficacy. Overprotective parenting may limit a child’s experiential learning and independent stress management [62]. This potentially affects the development and functioning of neural circuits essential for stress regulation and emotional control [71] and reduces brain plasticity. The effectiveness of CBT may depend on whether these neural pathways can be improved or compensated for during therapy despite the profound impact of poor parenting styles. Besides, individuals with a background of high parental overprotection might perceive therapy as a greater stressor, leading to heightened stress and thereby cortisol responses [29, 30]. Mothers, often being the primary caregivers, spend more time with their children than fathers [72]. This increased exposure means that a mother’s parenting style could have a more profound influence on the child’s developmental outcomes [72, 73].

This study has several strengths including its longitudinal design, the examination of a well-characterized clinical sample, inclusion of a HC group, and the integration of comprehensive clinical and imaging data. However, some limitations must be acknowledged: First, the PBI, a retrospective self-report questionnaire, is prone to negative recall biases, potentially distorting its impact on subsequent mental health outcomes. Nonetheless, empirical evidence highlights the temporal stability of the PBI [74] and this study demonstrated robustness against outliers and multiple sensitivity checks. Second, the PBI captures only a limited range of caregiving and environmental factors influencing white matter development, excluding aspects like parental mental health, socioeconomic status, and exposure to chronic stress, which should be considered when interpreting the findings. Third, the inclusion of patients with different depressive diagnoses and partial remission adds heterogeneity to the patient group, potentially explaining findings of lower FA in depression relative to HC cross-sectionally neither at baseline or at follow-up, which is in contrast to findings from prior cross-sectional studies [7, 8, 14]. This heterogeneity, however, offers a more realistic representation of individuals in our healthcare system seeking CBT for depression. Future research should consider including a waitlist or active control group and larger subgroups differentiated by remission status to evaluate the effects of CBT, remission status, and therapy-independent fluctuations in depressive symptoms on FA changes. Fourth, six patients with acute dysthymia or adjustment disorder at baseline were included, though there is evidence suggesting that individuals with dysthymia may show less responsiveness to therapy [75]. However, our robustness checks indicate that the results remain consistent even when accounting for varying levels of symptom severity at baseline, or when excluding patients with dysthymia and adjustment disorder. Fifth, while the naturalistic study design poses challenges in controlling factors during CBT, it is noteworthy that the supervised psychotherapists in training adhered to established manuals and national care guidelines for depression, ensuring a higher level of comparability in the administered treatment. Sixth, while TBSS offers several advantages, it is limited by its inability to reconstruct individual fiber tracts. Future studies could benefit from integrating tractography-based analyses to complement TBSS. Lastly, the mediation analysis was conducted on a relatively small sample, which may have resulted in insufficient statistical power to detect significant effects in the other parenting dimensions.

Overall, this study underscores the significance of WM microstructural integrity in depression and suggests that changes in WM microstructural integrity could serve as a potential neural mechanism linked to symptom improvement after CBT. Moreover, the findings indicate that positive parenting styles—characterized by high levels of care and low levels of overprotection—are predictive of fewer depressive symptoms following CBT, mediated by WM microstructural integrity changes. The results highlight the relevance of parenting styles and related microstructural changes in treating depression with CBT.

## Supplementary information

Supplementary Material

## Data Availability

The data of this study are available on reasonable request from the corresponding author. The data are not publicly available due to privacy or ethical restrictions.
