# Supplementary material for: Longitudinal associations between white matter integrity, early life adversities, and treatment response following cognitive-behavioral therapy in depression
Source: Neuropsychopharmacology. 2025 Feb 26;50(6):1000–7. doi: 10.1038/s41386-025-02070-x (PMC12032135; doi:10.1038/s41386-025-02070-x)
Supplement: Supplementary file 1 — Supplementary Material [file 41386_2025_2070_MOESM1_ESM.doc]

**Supplementary Information**

[Supplement 1: Exclusion criteria and sample selection 1](#__RefHeading___Toc187424446)

[Exclusion criteria 1](#__RefHeading___Toc187424447)

[Sample selection 2](#__RefHeading___Toc187424448)

[Supplement 2: Cognitive-behavioral therapy 2](#__RefHeading___Toc187424449)

[Supplement 3: DTI data acquisition and preprocessing 3](#__RefHeading___Toc187424450)

[DTI data acquisition 3](#__RefHeading___Toc187424451)

[DTI data preprocessing 3](#__RefHeading___Toc187424452)

[Supplement 4: Longitudinal TBSS pipeline 3](#__RefHeading___Toc187424453)

[Supplement 5: Effects in MD, RD, and AD 4](#__RefHeading___Toc187424454)

[Methods 4](#__RefHeading___Toc187424455)

[Results 5](#__RefHeading___Toc187424456)

[Supplement 6: Comparison of model fits of fixed vs. random slopes LME models 6](#__RefHeading___Toc187424457)

[Supplement 7: Definition of remission status 6](#__RefHeading___Toc187424458)

[Supplement 8: Supplementary Tables 7](#__RefHeading___Toc187424459)

[Supplement 9: Robustness checks 12](#__RefHeading___Toc187424460)

[Correction for outliers 12](#__RefHeading___Toc187424461)

[Correction for non-linear age effects 13](#__RefHeading___Toc187424462)

[Correction for additional covariates at baseline 15](#__RefHeading___Toc187424463)

[Correction for clinical characteristics within patients 17](#__RefHeading___Toc187424464)

[Analyses excluding patients suffering from acute dysthymia or acute adjustment disorder at baseline 19](#__RefHeading___Toc187424465)

# Supplement 1: Exclusion criteria and sample selection

# Exclusion criteria

In general, the study included participants aged 18 to 65 years at baseline with Caucasian ancestry. General exclusion criteria for all participants included any neurological abnormalities, organic mental disorders, brain injuries, or contraindications for MRI. Initially, all patients met the DSM-IV criteria for a primary diagnosis of either an acute or partially remitted major depressive disorder (depression), confirmed through the Structured Clinical Interview for DSM-IV (SCID-I, [1]). Additional exclusion criteria for patients included diagnoses of bipolar disorder, psychotic disorder, or acute substance dependence. For healthy controls (HC), an exclusion criterion was any lifetime diagnosis of a mental disorder according to the SCID-I [1].

## **Sample selection**

The study sample was drawn from the Prevention and Intervention Neuroimaging Cohort (PINC), initially comprising *N*=689 data points from patients with psychiatric diagnoses or healthy individuals across various assessment time points. First, assessment time points not relevant to the current study were excluded (*n*=269 data points removed), followed by the exclusion of *n*=90 participants who yet lacked follow-up assessments after baseline (90 data points removed). Furthermore, *n*=18 patients with depression who did not undergo cognitive behavioral therapy (CBT) between baseline and follow-up were excluded, removing an additional 36 data points. From the remaining group *n*=71 HC and *n*=76 patients with data at baseline and follow-up, *n*=4 HC who developed a diagnosis during the study interval were excluded (8 data points removed), along with 2 patients who had a fully remitted depression at baseline (4 data points removed). Additionally, *n*=3 patients who underwent more than 30 CBT sessions were excluded (6 data points removed), and 8 participants (6 patients with depression and 2 HC) were excluded due to unavailable diffusion tensor imaging (DTI) images at either baseline, follow-up, or both, or because their brain structural images failed data quality analysis [2], 16 data points removed). This resulted in a final study sample of *n*=65 patients with depression and *n*=65 HC, totalling 260 data points (130 each for baseline and follow-up).

# Supplement 2: Cognitive-behavioral therapy

CBT seeks to identify and modify maladaptive thinking and emotion processing patterns, while simultaneously fostering behavioral activation [3]. Patients in this study received 20 sessions of CBT (*M*=20.83, *SD*=3.70). These sessions were conducted by psychotherapists in training under the supervision of expert psychotherapists. Although naturalistic, the therapeutic interventions were generally consistent with established manuals for depressive disorders and adhered to the national care guidelines for unipolar depression [4], including techniques such as behavioral activation and cognitive restructuring.

# Supplement 3: DTI data acquisition and preprocessing

## **DTI data acquisition**

DTI data were obtained using 5 non-diffusion weighted images and 2 × 30 diffusion weighted images (b=1000sec/mm²) with a 3T whole body MRI scanner (Prisma, Siemens, Erlangen, Germany; GRAPPA acceleration factor of 2, 56 axial slices, 2.5mm thick with no gap, isotropic voxel size 2.5 × 2.5 × 2.5mm³, TE=90ms, TR=7300ms). In order to ensure data quality, the open-source software DTIPrep was used [5]. Motion artifacts were minimized by positioning participants in a supine orientation with additional padding during the scan.

## **DTI data preprocessing**

Preprocessing of DTI data was performed in FSL6.0.1 (http://fsl.fmrib.ox.ac.uk/fsl/fslwiki/, FMRIB, Oxford Center for Functional MRI of the Brain, University of Oxford, Department of Clinical Neurology, John Radcliffe Hospital, Oxford, United Kingdom) [6–8]. The b-vectors were rotated after correction for eddy and motion artifacts using FSL’s “eddy” [9]. Automated skull stripping was applied using FSL's brain extraction tool (BET) [10]. The first b0 image was used as the reference for image alignment. The diffusion tensor was computed using “DTIFIT” within FMRIB’s Diffusion Toolbox (FDT), providing estimates for fractional anisotropy (FA), mean diffusivity (MD), radial diffusivity (RD), and axial diffusivity (AD) [11]. FA represents the normalized variance of the three eigenvalues about their mean, indicating directional diffusion. MD is the average of all eigenvalues, while AD reflects primary diffusion direction and tract orientation, and RD describes motion perpendicular to the tract [12].

# Supplement 4: Longitudinal TBSS pipeline

The present longitudinal TBSS pipeline was adapted from others [13–15], incorporating the following steps:

1. FA-images were first registered (FLIRT) using an optimization scheme that has been validated for longitudinal studies [16,17]. The volumes at both time points were resampled halfway between the two time points, requiring only a single registration per volume and minimizing registration bias at either time point [18].
2. Both halfway-registered FA maps were averaged to create a subject-based template.
3. TBSS was used to automatically non-linearly register each subject’s FA template to the FMRIB58_FA standard-space (1x1x1mm³ Montreal Neurological Institute (MNI) standard space). All images were resliced to 1x1x1mm³ image space.
4. The mean FA image was created and thinned into a mean FA skeleton representing the centers of all tracts commonly occurring in the sample.
5. A threshold of FA>0.2 was applied to the skeleton. This binarization serves to suppress areas of low mean FA and/or high inter-subject variability.
6. The aligned FA images of each participant from both time-points were projected onto the skeleton.

Cluster size and peak voxel MNI coordinates were retrieved using FSL’s “cluster” tool. Anatomical labels of significant clusters corresponding to the “JHU White-Matter Labels” [19] were extracted using FSL’s “atlasquery” command. The average FA per participant of significant clusters was extracted for scatterplots and further analyses in *R* using FSL’s “fslstats”. Effect sizes were estimated with semi-partial *r*^2^ (*sr* ^2^) or partial eta^2^ (*n*^2^_p_) as described in Nakagawa and Schielzeth [20]. Analyses were repeated for MD, AD, and RD. The total intracranial volume (TIV) was obtained from T1-weighted (T1w) images using deepmriprep, a neural network-based preprocessing pipeline for T1w images developed in our workgroup [21].

# Supplement 5: Effects in MD, RD, and AD

## **Methods**

In the same manner as for FA, the other three DTI metrics (MD, RD, AD) were preprocessed and analyzed using the longitudinal TBSS pipeline implemented in FSL. For all analyses, the same covariates as for FA analyses were included. TFCE with 5000 permutations per test and a FWE-corrected threshold of *p*<.05 (two-sided when based on non-directional hypotheses and one-sided when based on directional hypotheses) was performed to adjust for multiple comparisons. Separate random intercepts fixed slopes LME models with MD, RD, or AD as outcome variables and time and diagnosis as predictor variables were performed to examine whether WM integrity changes differ between depression and HC over time (*analysis 1*). The main effect of time and the diagnosis×time interaction were analyzed. For cross-sectional analyses, group differences in MD, RD, and AD were analyzed using separate general models for baseline and follow-up. To investigate whether WM integrity changes are associated with symptom changes in patients (*analysis 2*), further random intercepts fixed slopes LME models were performed in patients with MD, RD, or AD as outcome variables and time and HDRS as predictor variables. The HDRS×time interaction was analyzed. Lastly, mediation models were performed in patients using a bootstrapping approach implemented in the macro PROCESS (http://www.processmacro.org) to examine whether parenting style is associated with symptom improvement after CBT and whether this association is mediated by WM integrity changes (*analysis 3*). Parenting style was entered as predictor variable, mean ΔMD, ΔRD, or ΔAD derived from significant clusters of *analysis 2* as mediator, and HDRS at follow-up as outcome variable into the model. Separate mediation models were calculated for each of the four PBI scores (maternal care, paternal care, maternal overprotection, paternal overprotection), with false discovery rate (FDR) correction applied to correct for multiple testing.

## **Results**

No significant main effect of time emerged for MD, RD, or AD (*p*_tfce-FWE_≥.121). However, a significant diagnosis×time interaction in the corpus callosum, superior longitudinal fasciculus, and corona radiata for MD (*p*_tfce-FWE_=.002, *sr*^2^=0.006, k=15203 voxel in 9 clusters, Tables S1-2), RD (*p*_tfce-FWE_=.003, *sr*^2^=0.012, k=11016 voxel in 9 clusters, Tables S1-2), and AD (*p*_tfce-FWE_=.013, *sr*^2^=0.029, k=811 voxel in 1 cluster, Tables S1-2) was found. Post-hoc-*t*-contrasts showed that patients with depression showed greater increases in MD (*p*_tfce-FWE_=.002, *sr*^2^=0.011, k=30746 voxel in 1 cluster, Tables S1-2) and RD (*p*_tfce-FWE_=.004, *sr*^2^=0.012, k=16498 voxel in 5 clusters, Tables S1-2), and decreases in AD (*p*_tfce-FWE_=.042, *sr*^2^=0.037, k=184 voxel in 2 clusters, Tables S1-2) over time compared to HC. Neither at baseline (*p*_tfce-FWE_≥.376), nor at follow-up (*p*_tfce-FWE_≥.062), cross-sectional group differences for MD, RD, or AD could be observed. Neither for MD (*p*_tfce-FWE_=.364), nor RD (*p*_tfce-FWE_=.126), nor AD (*p*_tfce-FWE_=.300), a significant HDRS×time interaction emerged. Therefore, no mediation analyses were performed for these DTI metrics.

# Supplement 6: Comparison of model fits of fixed vs. random slopes LME models

To ensure that the chosen modeling approach was appropriate, we compared the fit of LME models with random slopes to the simpler random intercept-only models for all LME analyses. Specifically, likelihood ratio tests (LRTs) were performed to evaluate whether the inclusion of random slopes significantly improved model fit. The results indicated no significant improvement in model fit with the inclusion of random slopes in any analysis (symptom improvement in patients with MDD following CBT: LRT *p*=.688, *analysis 1,* differences in FA changes between patients with depression and HC: LRT *p*=.103; *analysis 2*, the relationship between FA changes and symptom changes following CBT: LRT *p*=.166).

# Supplement 7: Definition of remission status

The diagnosis of patients as well as their remission status were determined using the DSM-IV criteria. Importantly, we included only patients with acute dysthymia and acute adjustment disorder at baseline, while patients diagnosed with MDD could be either acute or partially remitted at baseline.

**Major depressive disorder**: A current depressive episode was defined by the presence of five or more symptoms, occurring almost daily for at least two weeks, and significantly impairing daily life. At least one symptom had to be depressed mood or loss of interest/pleasure, with additional symptoms including: changes in sleep (insomnia or hypersomnia) and appetite, psychomotor agitation or retardation, fatigue or loss of energy, feelings of worthlessness or guilt, difficulty concentrating or making decisions, and recurrent thoughts of death or suicide. Partial remission was defined as either (1) persistence of some symptoms after a depressive episode, without meeting full DSM-IV criteria for a depressive episode, or (2) absence of depressive symptoms for a period shorter than two months. Complete remission was defined as the absence of symptoms for at least two months.

**Dysthymia:** A diagnosis of current dysthymia requires symptoms of depressed mood that persist for most of the day for at least two years. At least two other symptoms are required, including: Loss of appetite or overeating, insomnia or hypersomnia, low energy or fatigue, low self-esteem, poor concentration or difficulty making decisions, and feelings of hopelessness. These symptoms should not fulfil the criteria for MDD during the two-year period. For dysthymia, partial remission was defined as the presence of residual symptoms that no longer met the full diagnostic criteria or a remission lasting less than two months. Complete remission was defined as the absence of symptoms for at least two months.

**Adjustment disorder**: The diagnosis of current adjustment disorder requires the presence of emotional or behavioural symptoms (in the case of this study, depressive symptoms) occurring within three months of an identifiable stressor and causing clinically significant impairment in daily functioning or disproportionate stress, without fulfilling diagnostic criteria for another depressive disorder. Partial remission was defined as the presence of some symptoms without the full diagnostic criteria being met. Complete remission was defined as the disappearance of all symptoms and the absence of significant functional impairment.

# Supplement 8: Supplementary Tables

| **Supplementary Table 1.** Cluster sizes and coordinate of the peak voxel of DTI results | | | | | | |
| --- | --- | --- | --- | --- | --- | --- |
|  |  |  | | **MNI** | | |
| **Analysis** | **contrast** | ***k*** | ***p*_tfce-FWE_** | **x** | **y** | **z** |
| **Analysis 1:** Diagnosis × time | | |  |  |  |  |
|  | FA: *F*-test | 1061 | .006 | -21 | -34 | 38 |
|  |  | 384 | .030 | -15 | -38 | 27 |
|  | FA: *t*-test | 2999 | .008 | -21 | -35 | 38 |
|  | MD: *F*-test | 14357 | .002 | -24 | -10 | 44 |
|  |  | 419 | .044 | -11 | 29 | -13 |
|  |  | 181 | .043 | -18 | 44 | 2 |
|  |  | 91 | .048 | -12 | 12 | -7 |
|  |  | 63 | .042 | 18 | 33 | -11 |
|  |  | 56 | .046 | -12 | 7 | 2 |
|  |  | 22 | .049 | 16 | -26 | 56 |
|  |  | 8 | .048 | 8 | -6 | -11 |
|  |  | 6 | .049 | 15 | -13 | 58 |
|  | MD: *t*-test | 30746 | .002 | -34 | -13 | 37 |
|  | RD: *F*-test | 10727 | .003 | -20 | -29 | 46 |
|  |  | 72 | .049 | -44 | -48 | 36 |
|  |  | 54 | .047 | -43 | -23 | 9 |
|  |  | 50 | .048 | -60 | -31 | 10 |
|  |  | 45 | .046 | -12 | 4 | 5 |
|  |  | 36 | .048 | -27 | -1 | 37 |
|  |  | 12 | .049 | -7 | -26 | 55 |
|  |  | 11 | .050 | -47 | 0 | -18 |
|  |  | 9 | .050 | -46 | -43 | 35 |
|  | RD: *t*-test | 16442 | .004 | -37 | -11 | 30 |
|  |  | 22 | .048 | 19 | 2 | 12 |
|  |  | 16 | .049 | 31 | -18 | 37 |
|  |  | 12 | .049 | 24 | 14 | 17 |
|  |  | 6 | .050 | 27 | 5 | 25 |
|  | AD: *F*-test | 811 | .013 | -8 | -24 | 25 |
|  | AD: *t*-test | 141 | .042 | -5 | -22 | 24 |
|  |  | 43 | .046 | -8 | 1 | 27 |
| **Analysis 2:** HDRS × time | |  |  |  |  |  |
|  | FA: *t*-test | 4741 | .023 | 17 | 20 | 43 |
| **Exploratory analysis:** responder vs. non-responder | | | | | | |
|  | FA changes | 614 | .026 | -14 | 24 | 20 |
|  | FA at baseline | 1165 | .020 | 20 | 32 | 17 |
|  | FA at follow-up | 24271 | .004 | 26 | -15 | 32 |
| **Abbreviations.** DTI=Diffusion-tensor imaging, *k*=voxel count, MNI=Montreal Neurological Institute, FA= fractional anisotropy, MD= mean diffusivity, RD= radial diffusivity, AD= axial diffusivity. | | | | | | |

| **Supplementary Table 2.** Anatomical locations of the “JHU White-Matter Labels Atlas” of the significant effects estimated with “autoaq”. The numbers represent the average probability of the mask of the significant cluster to be a member of the different labeled regions within the atlas | | | | |
| --- | --- | --- | --- | --- |
| **contrast** | **region** | **hemisphere** | **percentage** | |
| **Analysis 1:** Diagnosis × time | |  |  |  |
| FA: *F*-test | Body of corpus callosum | / | 5.32 | |
|  | Splenium of corpus callosum | / | 13.61 | |
|  | Superior corona radiata | L | 6.51 | |
|  | Posterior corona radiata | L | 3.55 | |
| FA: *t*-test | Body of corpus callosum | / | 5.66 | |
|  | Splenium of corpus callosum | / | 13.75 | |
|  | Anterior corona radiata | L | 1.89 | |
|  | Superior corona radiata | L | 7.01 | |
|  | Posterior corona radiata | L | 3.77 | |
| MD: *F*-test | Genu of corpus callosum | / | 0.90 | |
|  | Body of corpus callosum | / | 5.69 | |
|  | Splenium of corpus callosum | / | 1.44 | |
|  | Anterior limb of internal capsule | L | 1.28 | |
|  | Posterior limb of internal capsule | L | 0.32 | |
|  | Retrolenticular part of internal capsule | L | 0.48 | |
|  | Anterior corona radiata | R | 0.21 | |
|  | Anterior corona radiata | L | 4.10 | |
|  | Superior corona radiata | L | 7.55 | |
|  | Posterior corona radiata | L | 2.29 | |
|  | Posterior thalamic radiation (incl. optic radiation) | L | 0.53 | |
|  | Sagittal stratum (incl. inferior longitidinal fasciculus and inferior fronto-occipital fasciculus) | L | 0.53 | |
|  | External capsule | L | 0.64 | |
|  | Cingulum | L | 0.11 | |
|  | Superior longitudinal fasciculus | L | 6.33 | |
|  | Uncinate fasciculus | L | 0.05 | |
| MD: *t*-test | Genu of corpus callosum | / | 0.44 | |
|  | Body of corpus callosum | / | 0.39 | |
|  | Splenium of corpus callosum | / | 0.31 | |
|  | Anterior limb of internal capsule | R | 0.75 | |
|  | Anterior limb of internal capsule | L | 1.20 | |
|  | Posterior limb of internal capsule | R | 0.10 | |
|  | Posterior limb of internal capsule | L | 0.81 | |
|  | Retrolenticular part of internal capsule | L | 0.36 | |
|  | Anterior corona radiata | R | 2.11 | |
|  | Anterior corona radiata | L | 2.97 | |
|  | Superior corona radiata | R | 2.08 | |
|  | Superior corona radiata | L | 4.22 | |
|  | Posterior corona radiata | R | 0.86 | |
|  | Posterior corona radiata | L | 1.43 | |
|  | Posterior thalamic radiation (incl. optic radiation) | L | 0.60 | |
|  | Sagittal stratum (incl. inferior longitidinal fasciculus and inferior fronto-occipital fasciculus) | L | 0.44 | |
|  | External capsule | R | 0.49 | |
|  | External capsule | L | 0.47 | |
|  | Cingulum | R | 0.96 | |
|  | Cingulum | L | 0.31 | |
|  | Superior longitudinal fasciculus | R | 1.72 | |
|  | Superior longitudinal fasciculus | L | 3.75 | |
|  | Superior fronto-occipital fasciculus | R | 0.34 | |
|  | Uncinate fasciculus | L | 0.03 | |
| RD: *F*-test | Body of corpus callosum | / | 0.29 | |
|  | Splenium of corpus callosum | / | 0.87 | |
|  | Anterior limb of internal capsule | L | 1.23 | |
|  | Posterior limb of internal capsule | L | 0.80 | |
|  | Retrolenticular part of internal capsule | L | 1.30 | |
|  | Anterior corona radiata | L | 2.39 | |
|  | Superior corona radiata | L | 9.62 | |
|  | Posterior corona radiata | L | 3.62 | |
|  | Posterior thalamic radiation (incl. optic radiation) | L | 0.65 | |
|  | Sagittal stratum (incl. inferior longitidinal fasciculus and inferior fronto-occipital fasciculus) | L | 0.58 | |
|  | External capsule | L | 0.58 | |
|  | Cingulum | L | 1.09 | |
|  | Superior longitudinal fasciculus | L | 7.59 | |
| RD: *t*-test | Genu of corpus callosum | / | 0.10 | |
|  | Body of corpus callosum | / | 0.73 | |
|  | Splenium of corpus callosum | / | 0.78 | |
|  | Anterior limb of internal capsule | R | 0.16 | |
|  | Anterior limb of internal capsule | L | 1.80 | |
|  | Posterior limb of internal capsule | L | 1.90 | |
|  | Retrolenticular part of internal capsule | L | 0.92 | |
|  | Anterior corona radiata | R | 0.05 | |
|  | Anterior corona radiata | L | 4.57 | |
|  | Superior corona radiata | L | 7.00 | |
|  | Posterior corona radiata | L | 2.63 | |
|  | Posterior thalamic radiation (incl. optic radiation) | L | 0.83 | |
|  | Sagittal stratum (incl. inferior longitidinal fasciculus and inferior fronto-occipital fasciculus) | L | 0.78 | |
|  | External capsule | L | 0.92 | |
|  | Cingulum | L | 1.02 | |
|  | Superior longitudinal fasciculus | R | 0.10 | |
|  | Superior longitudinal fasciculus | L | 6.13 | |
|  | Superior fronto-occipital fasciculus | L | 0.05 | |
|  | Uncinate fasciculus | L | 0.05 | |
| AD: *F*-test | Body of corpus callosum | / | 64.10 | |
|  | Splenium of corpus callosum | / | 33.98 | |
| AD: *t*-test | Body of corpus callosum | / | 100 | |
| **Analysis 2:** HDRS × time | |  |  | |
| FA: *t*-test | Genu of corpus callosum | / | 5.90 | |
|  | Body of corpus callosum | / | 3.99 | |
|  | Anterior limb of internal capsule | R | 8.61 | |
|  | Posterior limb of internal capsule | R | 0.32 | |
|  | Anterior corona radiata | R | 12.76 | |
|  | Superior corona radiata | R | 3.67 | |
|  | External capsule | R | 7.02 | |
|  | Superior longitudinal fasciculus | R | 6.06 | |
|  | Superior fronto-occipital fasciculus | R | 1.28 | |
| **Exploratory analysis:** responder vs. non-responder | | | | |
| FA changes | Genu of corpus callosum | / | 32.88 | |
|  | Body of corpus callosum | / | 30.14 | |
|  | Anterior corona radiata | L | 34.62 | |
| FA baseline | Body of corpus callosum | / | 2.99 | |
|  | Anterior corona radiata | R | 20.15 | |
|  | Superior corona radiata | R | 40.30 | |
|  | Posterior corona radiata | R | 2.34 | |
|  | Superior longitudinal fasciculus | R | 9.70 | |
| FA follow-up | Middle cerebellar peduncle | / | 0.07 | |
|  | Genu of corpus callosum | / | 2.12 | |
|  | Body of corpus callosum | / | 6.37 | |
|  | Splenium of corpus callosum | / | 2.22 | |
|  | Fornix (column and body of fornix) | / | 0.07 | |
|  | Corticospinal tract | R | 0.10 | |
|  | Corticospinal tract | L | 0.20 | |
|  | Cerebral peduncle | R | 0.91 | |
|  | Cerebral peduncle | L | 0.72 | |
|  | Anterior limb of internal capsule | R | 1.34 | |
|  | Anterior limb of internal capsule | L | 0.56 | |
|  | Posterior limb of internal capsule | R | 2.55 | |
|  | Posterior limb of internal capsule | L | 2.45 | |
|  | Retrolenticular part of internal capsule | R | 1.27 | |
|  | Retrolenticular part of internal capsule | L | 0.72 | |
|  | Anterior corona radiata | R | 2.78 | |
|  | Anterior corona radiata | L | 1.76 | |
|  | Superior corona radiata | R | 3.72 | |
|  | Superior corona radiata | L | 4.37 | |
|  | Posterior corona radiata | R | 1.40 | |
|  | Posterior corona radiata | L | 1.11 | |
|  | Posterior thalamic radiation (include optic radiation) | R | 0.13 | |
|  | Posterior thalamic radiation (include optic radiation) | L | 0.29 | |
|  | Sagittal stratum (include inferior longitudinal fasciculus and inferior fronto-occipital fasciculus) | R | 0.65 | |
|  | Sagittal stratum (include inferior longitudinal fasciculus and inferior fronto-occipital fasciculus) | L | 0.69 | |
|  | External capsule | R | 1.21 | |
|  | External capsule | L | 2.25 | |
|  | Fornix (cres) / Stria terminalis (cannot be resolved with current resolution) | R | 0.26 | |
|  | Fornix (cres) / Stria terminalis (cannot be resolved with current resolution) | L | 0.29 | |
|  | Superior longitudinal fasciculus | R | 3.69 | |
|  | Superior longitudinal fasciculus | L | 2.32 | |
|  | Superior fronto-occipital fasciculus (could be a part of anterior internal capsule) | R | 0.36 | |
|  | Uncinate fasciculus | R | 0.33 | |
|  | Tapetum | R | 0.10 | |
|  | Tapetum | L | 0.07 | |
| **Abbreviations.** L= Left, R= Right, FA= fractional anisotropy, MD= mean diffusivity, RD= radial diffusivity, AD= axial diffusivity. | | | | |

# Supplement 9: Robustness checks

## **Correction for outliers**

To ensure the robustness of the present results and mitigate the influence of potential outliers, the analyses in *R* using random intercept fixed slopes LMEs were recalculated, following the exclusion of outliers. Initially, the residuals from each LME model were extracted and *z*-standardized. Subsequently, outliers were defined by selecting all observations whose standardized residuals exceeded ±2*SD*. These identified outliers were then removed from the dataset, resulting in a cleaned dataset for each analysis. The results of these analyses are presented in **Supplementary** **Tables 3-4**. The greater decrease in FA in patients with depression compared to HC (*analysis 1*), as well as the association between symptom improvement and FA increases in patients with depression (*analysis 2*) remained significant after removing outliers. For outlier correction in the mediation analysis (*analysis 3*), a GLM was performed with maternal overprotection as the predictor variable and HDRS at follow-up as the independent variable, while correcting for HDRS_baseline_, TIV_baseline_, ΔTIV, age_baseline_, and interscan interval. Outliers were again defined by selecting all observations whose standardized residuals exceeded ±2*SD* and the mediation analysis was recalculated excluding *n*=3 outliers. Again, a positive association between maternal overprotection and HDRS at follow-up (*β*=0.421, SE=0.083, *t*=3.67, *p*<.001) was found. Further, ΔFA was negatively associated with HDRS at follow-up (*β*=-0.500, SE=71.348, *t*=-4.87, *p*<.001). Furthermore, a significant positive indirect (mediated) effect of maternal overprotection on HDRS at follow-up through ΔFA (*β*=0.202, SE=0.071, *p*=.008, 95%-CI [0.065, 0.343]) was observed. Lastly, a direct effect of maternal overprotection on HDRS at follow-up emerged (*β*=0.218, SE=0.076, *p*=.041, 95%-CI [0.006, 0.218]).

| **Supplementary Table 3*.*** The random intercept fixed slopes linear mixed model of *analysis* *1* was recalculated with FA as outcome variable, time (baseline vs. follow-up) and diagnosis (patients with depression vs. healthy controls) and their interaction as predictor variables, and age and TIV as covariates, while excluding 4 outliers with >2SD. | | | | |
| --- | --- | --- | --- | --- |
|  | **Estimates** | ***t*-value** | ***p*-value** | **sr^2^** |
| **Fixed effects** |  |  |  |  |
| (Intercept) | 0.560 | 32.73 | <.001 | 0.247 |
| Diagnosis×time | 0.002 | 7.27 | <.001 | 0.009 |
| Time | <0.001 | <0.01 | .999 | 0.000 |
| Diagnosis | 0.001 | 0.71 | .478 | 0.004 |
| Age | -0.001 | -5.99 | <.001 | 0.223 |
| TIV | <0.001 | 0.42 | .676 | 0.001 |
| **Random effects** |  |  |  |  |
| Intercept variance s_0_^2^ | 0.016 |  |  |  |
| Residual variance σ^2^ | 0.003 |  |  |  |
| **Abbreviations.** FA = fractional anisotropy, TIV=total intracranial volume. | | | | |

| **Supplementary Table 4***.* The random intercept fixed slopes linear mixed model of *analysis* *2* was recalculated in patients with FA as outcome variable, time (baseline vs. follow-up) and HDRS and their interaction as predictor variables, and age and TIV as covariates, while excluding 2 outliers with >2SD | | | | |
| --- | --- | --- | --- | --- |
|  | **Estimates** | ***t*-value** | ***p*-value** | **sr^2^** |
| **Fixed effects** |  |  |  |  |
| (Intercept) | 0.488 | 19.67 | <.001 | 0.043 |
| HDRS×time | -0.001 | -7.74 | <.001 | 0.030 |
| Time | 0.006 | 4.30 | <.001 | 0.011 |
| HDRS | <-0.001 | -0.67 | .501 | <0.001 |
| Age | >-0.001 | -0.97 | .334 | 0.001 |
| TIV | <0.001 | 1.23 | .220 | 0.022 |
| **Random effects** |  |  |  |  |
| Intercept variance s_0_^2^ | 0.018 |  |  |  |
| Residual variance σ^2^ | 0.004 |  |  |  |
| **Abbreviations.** FA=fractional anisotropy, TIV=total intracranial volume, HDRS=sum score of the Hamilton Depression Rating Scale. | | | | |

## **Correction for non-linear age effects**

To verify that our results were not affected by non-linear age effects, main analyses were recalculated including age^2^ as a further covariate. The greater decrease in FA in patients with depression compared to HC (*analysis 1*), as well as the association between symptom improvement and FA increases in patients with depression (*analysis 2*) remained significant after additionally controlling for age^2^ (**Supplementary** **Tables 5-6)**. Again, a positive association between maternal overprotection and HDRS at follow-up (*β*=0.408, SE=0.082, *t*=3.61, *p*<.001) was found. Further, ΔFA was negatively associated with HDRS at follow-up (*β*=-0.485, SE=68.825, *t*=-4.84, *p*<.001). Furthermore, a significant positive indirect (mediated) effect of maternal overprotection on HDRS at follow-up through ΔFA (*β*=0.179, SE=0.072, *p*=.014, 95%-CI [0.035, 0.321]) was observed. Lastly, a direct effect of maternal overprotection on HDRS at follow-up emerged (*β*=0.229, SE=0.074, *p*=.028, 95%-CI [0.017, 0.316]).

| **Supplementary Table 5*.*** The random intercept fixed slopes linear mixed model of *analysis* *1* was recalculated with FA as outcome variable, time (baseline vs. follow-up) and diagnosis (patients with depression vs. healthy controls) and their interaction as predictor variables, and age, TIV as well as age^2^ as covariates. | | | | |
| --- | --- | --- | --- | --- |
|  | **Estimates** | ***t*-value** | ***p*-value** | **sr^2^** |
| **Fixed effects** |  |  |  |  |
| (Intercept) | 0.556 | 25.82 | <.001 | 0.265 |
| Diagnosis×time | 0.002 | 7.07 | <.001 | 0.011 |
| Time | <0.000 | -0.63 | .468 | 0.000 |
| Diagnosis | 0.001 | 0.64 | .478 | 0.003 |
| Age | -0.001 | -0.66 | .491 | 0.003 |
| Age^2^ | <0.000 | -0.20 | .162 | 0.000 |
| TIV | <0.000 | 0.49 | .377 | 0.002 |
| **Random effects** |  |  |  |  |
| Intercept variance s_0_^2^ | 0.016 |  |  |  |
| Residual variance σ^2^ | 0.003 |  |  |  |
| **Abbreviations.** FA = fractional anisotropy, TIV=total intracranial volume. | | | | |

| **Supplementary Table 6***.* The random intercept fixed slopes linear mixed model of *analysis* *2* was recalculated in patients with FA as outcome variable, time (baseline vs. follow-up) and HDRS and their interaction as predictor variables, and TIV, age as well as age^2^ as covariates. | | | | |
| --- | --- | --- | --- | --- |
|  | **Estimates** | ***t*-value** | ***p*-value** | **sr^2^** |
| **Fixed effects** |  |  |  |  |
| (Intercept) | 0.488 | 19.69 | <.001 | 0.054 |
| HDRS×time | -0.001 | -7.08 | <.001 | 0.035 |
| Time | <0.000 | 1.64 | .893 | 0.011 |
| HDRS | -0.006 | -1.37 | .825 | 0.004 |
| Age | <0.000 | 0.14 | .113 | 0.000 |
| Age^2^ | <0.000 | -0.40 | .308 | 0.000 |
| TIV | <0.000 | 1.31 | 0805 | 0.025 |
| **Random effects** |  |  |  |  |
| Intercept variance s_0_^2^ | 0.017 |  |  |  |
| Residual variance σ^2^ | 0.004 |  |  |  |
| **Abbreviations.** FA=fractional anisotropy, TIV=total intracranial volume, HDRS=sum score of the Hamilton Depression Rating Scale. | | | | |

## **Correction for additional covariates at baseline**

We have not previously corrected for baseline covariates in longitudinal analyses, as they would be treated as constant values in the model that are already taken into account by the random intercept of participants. However, to show that our results remain significant, the main longitudinal analyses were repeated and additionally controlled for baseline FA (*analysis 1*) as well as baseline FA and baseline HDRS (*analysis 2*). These results showed that even when correcting for these baseline values, the decline in FA in patients with MDD following CBT compared to HC over time (*analysis 1*, **Supplementary Table 7**) as well as the association between FA increases and symptom improvement over time in patients (*analysis 2*, **Supplementary Table 8**) could be re-established.

| **Supplementary Table 7*.*** The random intercept fixed slopes linear mixed model of *analysis* *1* was recalculated with FA as outcome variable, time (baseline vs. follow-up) and diagnosis (patients with depression vs. healthy controls) and their interaction as predictor variables, and age, TIV as well as FA at baseline as covariates. | | | | |
| --- | --- | --- | --- | --- |
|  | **Estimates** | ***t*-value** | ***p*-value** | **sr^2^** |
| **Fixed effects** |  |  |  |  |
| (Intercept) | 0.029 | 3.36 | <.001 | 0.959 |
| Diagnosis×time | 0.002 | 7.81 | <.001 | 0.195 |
| Time | >-0.001 | -1.10 | .272 | 0.005 |
| Diagnosis | 0.002 | 6.56 | <.001 | 0.146 |
| Age | -<0.001 | -0.80 | .423 | 0.003 |
| TIV | <0.001 | 0.55 | .580 | 0.001 |
| FA at baseline | 0.995 | 66.50 | <.001 | 0.946 |
| **Random effects** |  |  |  |  |
| Intercept variance s_0_^2^ | 0.000 |  |  |  |
| Residual variance σ^2^ | 0.004 |  |  |  |
| **Abbreviations.** FA = fractional anisotropy, TIV=total intracranial volume. | | | | |

| **Supplementary Table 8***.* The random intercept fixed slopes linear mixed model of *analysis 2* was recalculated in patients with FA as outcome variable, time (baseline vs. follow-up) and HDRS and their interaction as predictor variables, and age, TIV, FA at baseline, and HDRS at baseline as covariates. | | | | |
| --- | --- | --- | --- | --- |
|  | **Estimates** | ***t*-value** | ***p*-value** | **sr^2^** |
| **Fixed effects** |  |  |  |  |
| (Intercept) | 0.035 | 2.94 | .005 | 0.949 |
| HDRS×time | -0.001 | -8.34 | <.001 | 0.365 |
| Time | 0.008 | 5.57 | <.001 | 0.204 |
| HDRS | <-0.001 | -1.69 | .097 | 0.023 |
| Age | >-0.001 | -2.16 | .035 | 0.037 |
| TIV | <0.001 | 1.41 | .165 | 0.016 |
| FA at baseline | 0.935 | 43.86 | <.001 | 0.941 |
| HDRS at baseline | -0.001 | -4.46 | <.001 | 0.141 |
| **Random effects** |  |  |  |  |
| Intercept variance s_0_^2^ | 0.000 |  |  |  |
| Residual variance σ^2^ | 0.004 |  |  |  |
| **Abbreviations.** FA=fractional anisotropy, TIV=total intracranial volume, HDRS=sum score of the Hamilton Depression Rating Scale. | | | | |

Furthermore, with regard to our mediation analysis (i.e., *analysis 3*), where we included the change score of FA as a mediator variable (here we did not include a random intercept), the inclusion of baseline covariates makes more sense. Therefore, we performed a robustness check, including baseline FA levels as a further covariate. Again, a positive association between maternal overprotection and HDRS at follow-up (*β*=0.304, SE=0.082, *t*=3.70, *p*<.001) was found. Further, ΔFA was negatively associated with HDRS at follow-up (*β*=-0.533, SE=68.376, *t*=-5.35, *p*<.001). Furthermore, a significant positive indirect (mediated) effect of maternal overprotection on HDRS at follow-up through ΔFA (*β*=0.208, SE=0.079, *p*=.007, 95%-CI [0.050, 0.367]) was observed. Lastly, a direct effect of maternal overprotection on HDRS at follow-up emerged (*β*=0.202, SE=0.073, *p*=.041, 95%-CI [0.006, 0.299]).

In addition, in order to control for differences in symptom severity between different depressive diagnoses and their influence on response, we repeated the exploratory analysis comparing responder vs. non-responder, further adjusting for the HDRS-21 total score at baseline as a continuous measure of depressive symptom severity. Even with this adjustment, the findings remain consistent: responder exhibit a greater increase in FA over time compared to non-responder (**Supplementary Table 9**). Additionally, responder show higher FA at both baseline (**Supplementary Table 10**) and follow-up (**Supplementary Table 11**) compared to non-responder.

| **Supplementary Table 9*.*** The random intercept fixed slopes linear mixed model was recalculated in patients with FA as outcome variable, time (baseline vs. follow-up) and response (yes vs. no) and their interaction as predictor variables, and age, TIV, remission status at baseline, and HDRS at baseline as covariates. | | | | |
| --- | --- | --- | --- | --- |
|  | **Estimates** | ***t*-value** | ***p*-value** | **sr^2^** |
| **Fixed effects** |  |  |  |  |
| (Intercept) | 0.575 | 10.69 | <.001 | 0.036 |
| Response × time | 0.005 | 4.99 | <.001 | 0.011 |
| Time | 0.001 | 0.35 | .731 | 0.000 |
| Response | 0.005 | 0.91 | .366 | 0.010 |
| Age | >-0.001 | -1.45 | .152 | 0.002 |
| TIV | <0.001 | 0.77 | .447 | 0.009 |
| Remission status at baseline | -0.001 | -0.66 | .511 | 0.000 |
| HDRS at baseline | >-0.001 | -0.27 | .791 | 0.001 |
| **Random effects** |  |  |  |  |
| Intercept variance s_0_^2^ | 0.032 |  |  |  |
| Residual variance σ^2^ | 0.007 |  |  |  |
| **Abbreviations.** FA = fractional anisotropy, TIV=total intracranial volume, HDRS=sum score of the Hamilton Depression Rating Scale. | | | | |

| **Supplementary Table 10***.* The general linear model was recalculated in patients with FA at baseline as outcome variable and response (yes vs. no) as predictor variable, and age, TIV, remission status at baseline, and HDRS at baseline as covariates. | | | | |
| --- | --- | --- | --- | --- |
|  | **Estimates** | ***t*-value** | ***p*-value** | **sr^2^** |
| **Fixed effects** |  |  |  |  |
| (Intercept) | 0.548 | 11.94 | <.001 | 0.297 |
| Response | 0.019 | 4.00 | <.001 | 0.213 |
| Age | -0.001 | -0.98 | .332 | 0.016 |
| TIV | <0.001 | -0.40 | .691 | 0.003 |
| Remission status at baseline | -0.019 | -2.14 | .037 | 0.072 |
| HDRS at baseline | -0.001 | -0.93 | .355 | 0.015 |
| **Abbreviations.** FA=fractional anisotropy, TIV=total intracranial volume, HDRS=sum score of the Hamilton Depression Rating Scale. | | | | |

| **Supplementary Table 11***.* The general linear model was recalculated in patients with FA at follow-up as outcome variable and response (yes vs. no) as predictor variable, and age at follow-up, TIV at follow-up, remission status at baseline, and HDRS at baseline as covariates. | | | | |
| --- | --- | --- | --- | --- |
|  | **Estimates** | ***t*-value** | ***p*-value** | **sr^2^** |
| **Fixed effects** |  |  |  |  |
| (Intercept) | 0.529 | 20.09 | <.001 | 0.403 |
| Response | 0.014 | 3.78 | <.001 | 0.195 |
| Age | <-0.001 | -2.91 | .005 | 0.126 |
| TIV | <0.001 | 1.51 | .137 | 0.037 |
| Remission status at baseline | <-0.001 | -0.18 | .858 | 0.001 |
| HDRS at baseline | <-0.001 | -0.31 | .759 | 0.002 |
| **Abbreviations.** FA=fractional anisotropy, TIV=total intracranial volume, HDRS=sum score of the Hamilton Depression Rating Scale. | | | | |

## **Correction for clinical characteristics within patients**

To verify that results of *analysis 2* and *analysis 3* are not simply the consequence of clinical differences in the sample of patients with depression, analyses were repeated while correcting for clinical characteristics. The presence of recurrent depressive episodes before study participation were collected at baseline using self-reports and validated by the SCID-I [1]. Furthermore, the presence of comorbidities was assessed during the SCID-I interview [1]. At both time points, information about the current medication intake using the Medication Load Index [22,23] was collected. Medications were classified as absent (0), low (1, equal to or below the average dose), or high (2, above the average dose) based on the midpoint of the daily dosage range recommended in the Physician’s Desk Reference [24]. Subsequently, a composite measure of total medication exposure for each participant at baseline was derived by summing the scores of all individual medications.

Overall, the association between FA increases and symptom improvement remained significant (**Supplementary Table 12**). When correcting for these clinical characteristics within the mediation analysis, a positive association between maternal overprotection and HDRS at follow-up (*β*=0.372, SE=0.084, *t*=3.21, *p*=.002) was found again. Further, ΔFA was negatively associated with HDRS at follow-up (*β*=-0.476, SE=70.892, *t*=-4.61, *p*<.001). Furthermore, a significant positive indirect (mediated) effect of maternal overprotection on HDRS at follow-up through ΔFA (*β*=0.164, SE=0.068, *p*=.023, 95%-CI [0.021, 0.295]) was observed. No direct effect of parental overprotection on HDRS at follow-up emerged (95%-CI [-0.001, 0.304]).

| **Supplementary Table 12***.* The random intercept fixed slopes linear mixed model of *analysis 2* was recalculated in patients with FA as outcome variable, time (baseline vs. follow-up) and HDRS and their interaction as predictor variables, as well as MedIndex, comorbidities (yes vs. no), and recurrent depressive episodes before baseline, on top of age and TIV, as covariates. | | | | |
| --- | --- | --- | --- | --- |
|  | **Estimates** | ***t*-value** | ***p*-value** | **sr^2^** |
| **Fixed effects** |  |  |  |  |
| (Intercept) | 0.483 | 20.04 | <.001 | 0.073 |
| HDRS×time | -0.001 | -6.79 | <.001 | 0.037 |
| Time | 0.007 | 3.92 | <.001 | 0.015 |
| HDRS | -0.002 | -1.77 | .081 | 0.005 |
| Age | -0.001 | -1.40 | .164 | 0.002 |
| TIV | <0.001 | 1.38 | .170 | 0.028 |
| MedIndex | >-0.001 | -0.65 | .516 | 0.001 |
| Comorbidities | 0.003 | 0.99 | .324 | 0.011 |
| Recurrent depressive episodes | 0.002 | 0.87 | .383 | 0.005 |
| **Random effects** |  |  |  |  |
| Intercept variance s_0_^2^ | 0.016 |  |  |  |
| Residual variance σ^2^ | 0.004 |  |  |  |
| **Abbreviations.** FA = fractional anisotropy, TIV=total intracranial volume, MedIndex=medication load index, HDRS=sum score of the Hamilton Depression Rating Scale. | | | | |

## **Analyses excluding patients suffering from acute dysthymia or acute adjustment disorder at baseline**

To verify that the present results remain stable, even when including only patients with an acute or partially remitted depression in the patient sample, all main analyses were repeated excluding patients suffering from acute dysthymia or acute adjustment disorder at baseline (*n*=6). The results of these analyses are presented in **Supplementary Tables 13-14**. The greater decrease in FA in depression compared to HC (*analysis 1*), as well as the association between symptom improvement and FA increases in patients with depression (*analysis 2*) remained significant after excluding patients suffering from acute dysthymia or acute adjustment disorder at baseline. For *analysis 3*, a positive association between maternal overprotection and HDRS at follow-up (*β*=0.442, SE=0.090, *t*=3.57, *p*<.001) was found. Further, ΔFA was negatively associated with HDRS at follow-up (*β*=-0.494, SE=72.519, *t*=-4.57, *p*<.001). Furthermore, a significant positive indirect (mediated) effect of maternal overprotection on HDRS at follow-up through ΔFA (*β*=0.178, SE=0.079, *p*=.023, 95%-CI [0.023, 0.334]) was observed. Lastly, a direct effect of parental overprotection on HDRS at follow-up emerged (*β*=0.263, SE=0.081, *p*=.022, 95%-CI [0.028, 0.356]).

| **Supplementary Table 13***.* The random intercept fixed slopes linear mixed model of *analysis* *1* was recalculated with FA as outcome variable, time (baseline vs. follow-up) and diagnosis (patients with depression vs. healthy controls) and their interaction as predictor variables, and age and TIV as covariate, while excluding patients suffering from acute dysthymia or acute adjustment disorder at baseline. | | | | |
| --- | --- | --- | --- | --- |
|  | **Estimates** | ***t*-value** | ***p*-value** | **sr^2^** |
| **Fixed effects** |  |  |  |  |
| (Intercept) | 0.560 | 32.30 | <.001 | 0.262 |
| Diagnosis×time | 0.002 | 6.71 | <.001 | 0.009 |
| Time | <0.001 | -0.08 | .934 | 0.000 |
| Diagnosis | 0.001 | 0.67 | .503 | 0.004 |
| Age | -0.001 | -6.20 | <.001 | 0.227 |
| TIV | <0.001 | 0.38 | .704 | 0.001 |
| **Random effects** |  |  |  |  |
| Intercept variance s_0_^2^ | 0.017 |  |  |  |
| Residual variance σ^2^ | 0.004 |  |  |  |
| **Abbreviations.** FA = fractional anisotropy, TIV=total intracranial volume. | | | | |

| **Supplementary Table 14***.* The random intercept fixed slopes linear mixed model of *analysis* *2* was recalculated in patients with FA as outcome variable, time (baseline vs. follow-up) and HDRS and their interaction as predictor variables, and age and TIV as covariates, excluding patients suffering from acute dysthymia or acute adjustment disorder at baseline. | | | | |
| --- | --- | --- | --- | --- |
|  | **Estimates** | ***t*-value** | ***p*-value** | **sr^2^** |
| **Fixed effects** |  |  |  |  |
| (Intercept) | 0.498 | 20.52 | <.001 | 0.044 |
| HDRS×time | -0.001 | -6.44 | <.001 | 0.036 |
| Time | 0.007 | 3.88 | <.001 | 0.016 |
| HDRS | -0.002 | -1.29 | .202 | 0.003 |
| Age | >-0.001 | -1.36 | .176 | 0.002 |
| TIV | <0.001 | 0.87 | .383 | 0.012 |
| **Random effects** |  |  |  |  |
| Intercept variance s_0_^2^ | 0.022 |  |  |  |
| Residual variance σ^2^ | 0.005 |  |  |  |
| **Abbreviations.** FA=fractional anisotropy, TIV=total intracranial volume, HDRS=sum score of the Hamilton Depression Rating Scale. | | | | |

As an additional sensitivity check, we have now repeated the responder vs. non-responder exploratory analysis, excluding patients diagnosed with dysthymia or adjustment disorder. These results demonstrate that even after excluding n=6 patients with these diagnoses, responder still show a greater increase in FA over time compared to non-responder (**Supplementary Table 15**). Furthermore, responder exhibit higher FA levels at both baseline (**Supplementary Table 16**) and follow-up (**Supplementary Table 17**) compared to non-responder.

| **Supplementary Table 15*.*** The random intercept fixed slopes linear mixed model was recalculated in patients with MDD with FA as outcome variable, time (baseline vs. follow-up) and response (yes vs. no) and their interaction as predictor variables, and age, TIV, and remission status at baseline as covariates, excluding patients suffering from acute dysthymia or acute adjustment disorder at baseline. | | | | |
| --- | --- | --- | --- | --- |
|  | **Estimates** | ***t*-value** | ***p*-value** | **sr^2^** |
| **Fixed effects** |  |  |  |  |
| (Intercept) | 0.576 | 12.28 | <.001 | 0.034 |
| Response×time | 0.005 | 4.75 | <.001 | 0.010 |
| Time | 0.001 | 0.41 | .682 | 0.000 |
| Response | 0.005 | 0.91 | .364 | 0.009 |
| Age | >-0.001 | -1.69 | .097 | 0.002 |
| TIV | <0.001 | 0.67 | .504 | 0.008 |
| Remission status at baseline | 0.001 | 0.32 | .750 | 0.000 |
| **Random effects** |  |  |  |  |
| Intercept variance s_0_^2^ | 0.033 |  |  |  |
| Residual variance σ^2^ | 0.007 |  |  |  |
| **Abbreviations.** FA = fractional anisotropy, TIV=total intracranial volume, HDRS=sum score of the Hamilton Depression Rating Scale, MDD=major depressive disorder. | | | | |

| **Supplementary Table 16***.* The general linear model was recalculated in patients with FA at baseline as outcome variable and response (yes vs. no) as predictor variable, and age, TIV, remission status at baseline, and HDRS at baseline as covariates. | | | | |
| --- | --- | --- | --- | --- |
|  | **Estimates** | ***t*-value** | ***p*-value** | **sr^2^** |
| **Fixed effects** |  |  |  |  |
| (Intercept) | 0.548 | 11.94 | <.001 | 0.297 |
| Response | 0.019 | 4.00 | <.001 | 0.213 |
| Age | -0.001 | -0.98 | .332 | 0.016 |
| TIV | <0.001 | -0.40 | .691 | 0.003 |
| Remission status at baseline | -0.019 | -2.14 | .037 | 0.072 |
| HDRS at baseline | -0.001 | -0.93 | .355 | 0.015 |
| **Abbreviations.** FA=fractional anisotropy, TIV=total intracranial volume, HDRS=sum score of the Hamilton Depression Rating Scale. | | | | |

| **Supplementary Table 17***.* The general linear model was recalculated in patients with FA at follow-up as outcome variable and response (yes vs. no) as predictor variable, and age at follow-up, TIV at follow-up, remission status at baseline, and HDRS at baseline as covariates. | | | | |
| --- | --- | --- | --- | --- |
|  | **Estimates** | ***t*-value** | ***p*-value** | **sr^2^** |
| **Fixed effects** |  |  |  |  |
| (Intercept) | 0.529 | 20.09 | <.001 | 0.403 |
| Response | 0.014 | 3.78 | <.001 | 0.195 |
| Age | <-0.001 | -2.91 | .005 | 0.126 |
| TIV | <0.001 | 1.51 | .137 | 0.037 |
| Remission status at baseline | <-0.001 | -0.18 | .858 | 0.001 |
| HDRS at baseline | <-0.001 | -0.31 | .759 | 0.002 |
| **Abbreviations.** FA=fractional anisotropy, TIV=total intracranial volume, HDRS=sum score of the Hamilton Depression Rating Scale. | | | | |

**References**

1. Wittchen H-U, Wunderlich U, Gruschwitz S, Zaudig M. SKID I. Strukturiertes Klinisches Interview für DSM-IV. Achse I: Psychische Störungen. Interviewheft und Beurteilungsheft. Eine deutschsprachige, erweiterte Bearb. d. amerikanischen Originalversion des SKID I. 1997. 1997.

2. Vogelbacher C, Möbius TWD, Sommer J, Schuster V, Dannlowski U, Kircher T, et al. The Marburg-Münster Affective Disorders Cohort Study (MACS): A quality assurance protocol for MR neuroimaging data. Neuroimage. 2018;172:450–460.

3. Beck AT. Cognitive Therapy of Depression. Guilford Press; 1979.

4. Bundesärztekammer (BÄK), Kassenärztliche Bundesvereinigung (KBV), Arbeitsgemeinschaft der Wissenschaftlichen Medizinischen Fachgesellschaften (AWMF), editors. Nationale VersorgungsLeitlinie Unipolare Depression. BÄK, KBV, AWMF; 2022.

5. Oguz I, Farzinfar M, Matsui J, Budin F, Liu Z, Gerig G, et al. DTIPrep: quality control of diffusion-weighted images. Front Neuroinform. 2014;8:4.

6. Jenkinson M, Beckmann CF, Behrens TEJ, Woolrich MW, Smith SM. FSL. Neuroimage. 2012;62:782–790.

7. Smith SM, Jenkinson M, Woolrich MW, Beckmann CF, Behrens TEJ, Johansen-Berg H, et al. Advances in functional and structural MR image analysis and implementation as FSL. Neuroimage. 2004;23 Suppl 1:S208-219.

8. Woolrich MW, Jbabdi S, Patenaude B, Chappell M, Makni S, Behrens T, et al. Bayesian analysis of neuroimaging data in FSL. Neuroimage. 2009;45:S173-186.

9. Andersson JLR, Sotiropoulos SN. An integrated approach to correction for off-resonance effects and subject movement in diffusion MR imaging. NeuroImage. 2016;125:1063–1078.

10. Smith SM. Fast robust automated brain extraction. Human Brain Mapping. 2002;17:143–155.

11. Behrens TEJ, Woolrich MW, Jenkinson M, Johansen-Berg H, Nunes RG, Clare S, et al. Characterization and propagation of uncertainty in diffusion-weighted MR imaging. Magn Reson Med. 2003;50:1077–1088.

12. Alexander AL, Hurley SA, Samsonov AA, Adluru N, Hosseinbor AP, Mossahebi P, et al. Characterization of cerebral white matter properties using quantitative magnetic resonance imaging stains. Brain Connect. 2011;1:423–446.

13. Engvig A, Fjell AM, Westlye LT, Moberget T, Sundseth Ø, Larsen VA, et al. Memory training impacts short-term changes in aging white matter: a longitudinal diffusion tensor imaging study. Hum Brain Mapp. 2012;33:2390–2406.

14. Leenders AEM, Damatac CG, Soheili-Nezhad S, Chauvin RJM, Mennes MJJ, Zwiers MP, et al. Associations between attention-deficit hyperactivity disorder (ADHD) symptom remission and white matter microstructure: A longitudinal analysis. JCPP Advances. 2021;1:e12040.

15. Madhyastha T, Mérillat S, Hirsiger S, Bezzola L, Liem F, Grabowski T, et al. Longitudinal reliability of tract-based spatial statistics in diffusion tensor imaging. Hum Brain Mapp. 2014;35:4544–4555.

16. Jenkinson M, Smith S. A global optimisation method for robust affine registration of brain images. Med Image Anal. 2001;5:143–156.

17. Smith SM, Jenkinson M, Johansen-Berg H, Rueckert D, Nichols TE, Mackay CE, et al. Tract-based spatial statistics: voxelwise analysis of multi-subject diffusion data. Neuroimage. 2006;31:1487–1505.

18. Smith SM, De Stefano N, Jenkinson M, Matthews PM. Normalized accurate measurement of longitudinal brain change. J Comput Assist Tomogr. 2001;25:466–475.

19. Oishi K, Faria A, Jiang H, Li X, Akhter K, Zhang J, et al. Atlas-based whole brain white matter analysis using large deformation diffeomorphic metric mapping: Application to normal elderly and Alzheimer’s disease participants. Neuroimage. 2009;46:486–499.

20. Nakagawa S, Schielzeth H. A general and simple method for obtaining R2 from generalized linear mixed-effects models. Methods in Ecology and Evolution. 2013;4:133–142.

21. Fisch L, Winter NR, Goltermann J, Barkhau C, Emden D, Ernsting J, et al. deepmriprep: Voxel-based Morphometry (VBM) Preprocessing via Deep Neural Networks. arXiv. 2024. 2024. https://doi.org/10.48550/arXiv.2408.10656.

22. Hassel S, Almeida JR, Kerr N, Nau S, Ladouceur CD, Fissell K, et al. Elevated striatal and decreased dorsolateral prefrontal cortical activity in response to emotional stimuli in euthymic bipolar disorder: no associations with psychotropic medication load. Bipolar Disorders. 2008;10:916–927.

23. Redlich R, Almeida JR, Grotegerd D, Opel N, Kugel H, Heindel W, et al. Brain Morphometric Biomarkers Distinguishing Unipolar and Bipolar Depression: A Voxel-Based Morphometry–Pattern Classification Approach. JAMA Psychiatry. 2014;71:1222–1230.

24. PDR Network, Montvale, NJ. Physicians’ desk reference (71st ed., 2017). 2017. 2017.
